# Optimized strategy to mitigate daratumumab interference in blood bank testing: Reducing cost and time

**DOI:** 10.1093/ajcp/aqaf060

**Published:** 2025-07-02

**Authors:** Tom Yu, Antonio Insigne, Walia Anushka, Elena Nedelcu

**Affiliations:** University of California San Francisco, San Francisco, CA, United States; San Francisco State University, San Francisco, CA, United States; University of California San Francisco, San Francisco, CA, United States; University of California San Francisco, San Francisco, CA, United States

**Keywords:** daratumumab, pretransfusion testing interference, RBC alloimmunization, cost-effectiveness

## Abstract

**Objective:**

Drug interference with pretransfusion testing in patients with multiple myeloma (MM) treated with daratumumab (DARA) is well recognized. Current guidelines recommend that these patients should have a red blood cell (RBC) phenotype or genotype before DARA initiation; however, there are no other standards for pretransfusion testing. While prior publications report mitigation strategies and low RBC alloimmunization risk, they do not propose a cost-effective strategy for pretransfusion testing. This study aims to assess the RBC alloimmunization risk in patients treated with DARA and propose a cost-effective algorithm for pretransfusion testing.

**Methods:**

This is a retrospective study of patients treated with DARA and receiving RBC transfusions over 9.4 years (October 1, 2015, to January 30, 2025). Demographic data; a complete serologic profile, including blood typing, antibody screen (Ab screen), and antibody identification (Ab ID); RBC phenotype/genotype; and crossmatch data were collected for each patient. The clinically significant antibody formation incidence was recorded pre- and post-DARA and compared with a control group, with statistical significance defined as *P* < .05. The mitigation strategy initially used for pretransfusion testing and its optimized version are described along with their cost.

**Results:**

Of the 850 patients with MM on DARA therapy who were identified, 172 (20%) received at least 1 RBC transfusion. Ab screens were performed on all patients pre- and post-DARA therapy. Following drug administration, all patients showed a panagglutinin, and no patients formed new clinically significant alloantibodies. The turnaround time (TAT) and cost significantly decreased when the pretransfusion strategy with optimizing pretransfusion strategy.

**Conclusions:**

This is the most extensive study on patients treated with DARA and transfused, demonstrating no increased alloimmunization risk. DARA-related transfusion interference may be successfully mitigated by the novel strategy proposed here.

## INTRODUCTION

A known challenge in pretransfusion testing in patients with multiple myeloma (MM) treated with the anti-CD38 drug daratumumab (DARA) is DARA interference with indirect antiglobulin testing (IAT). This occurs due to DARA binding to CD38 expressed on the surface of red blood cells (RBCs). Several strategies to mitigate DARA-induced IAT panreactivity are in use. The most common of these methods is the pretreatment of RBCs with 0.2 mol/L dithiothreitol (DTT), which denatures CD38 by disrupting disulfide bonds.^1^ Treatment of RBCs with proteolytic enzymes, inhibition of RBC-DARA binding by F(ab′)2 fragments, and use of CD38-negative cord RBCs have also been proposed.^1-3^ Additionally, many institutions conduct universal phenotyping or genotyping before DARA initiation, and some transfuse with extended antigen-matched RBC units to reduce the risk of alloantibody formation.^4^ Still, universal standards for identifying clinically significant antibodies in patients receiving DARA have yet to be established.

Current practices to detect clinically significant antibodies in patients treated with DARA add substantial labor and cost to pretransfusion testing. In 1 study, the annual cost of a theoretical universal genotyping approach with the provision of phenotypically similar RBC transfusions was estimated to be $934.83 per patient receiving DARA.^5^ However, despite extensive efforts to prevent and detect alloantibody formation, increasing evidence suggests a low risk of RBC alloimmunization in patients treated with DARA, with rates ranging from 0% to 4% reported in prior studies.^6-8^ Given this, a more efficient strategy for pretransfusion testing of these patients should be considered. Further data on alloimmunization rates in patients receiving DARA can inform pretransfusion testing guidelines for this population.

In this study, we evaluate the risk of RBC alloimmunization in patients with MM receiving DARA, describe our experience with a pretransfusion testing optimization strategy and assess its cost-effectiveness, and propose a new algorithm for pretransfusion testing in this population.

## MATERIALS AND METHODS

This retrospective study was approved by our center’s institutional review board (#22-36170) and conducted in compliance with ethical guidelines, ensuring adherence to research protocols.

### Patient and control group, study period, and data collected

Patients with MM treated with DARA (Darzalex; Janssen-Cilag) who received RBC transfusions from October 1, 2015, to January 30, 2025, were included in the study. The study period started with the first dose of DARA and ended with the patient’s death or the latest Ab screen available for review. The control group was selected via admissions codes and comprised patients diagnosed with MM within the same time frame who received RBC transfusions but were not treated with DARA. Hospital encounters, demographics, blood testing results, transfusion history, and alloimmunization rate were compared with those of a control group who received transfusions within the same period.

### Overall strategy for pretransfusion testing

At our center, pretransfusion testing of patients with MM has been a routine part of our daily practice. The protocols evolved, as illustrated in **Table 1** and described below:

**Table 1:** Summary of Testing Strategy for Pretransfusion Testing on Patients Undergoing DARA Therapy

Steps	Phase I	Phase II
BB notification		✓
Automated blood typing	✓	✓
Automated Ab screen	✓	
Manual Ab screen	✓	✓
Prepare (0.2 mol/L DTT reagent RBC)	✓	
Prepare 7-day 0.2 mol/L DTT reagent RBC		✓
Repeat Ab screen with 0.2 mol/L DTT reagent RBC	✓	✓
Full phenotype	✓	
K or Kk typing		✓
RBC selection and crossmatching	✓	✓

The complete phenotype was determined via serology or predicted via genotype. Abbreviations: BB, blood bank; DARA, daratumumab; DTT, dithiothreitol; RBC, red blood cell.

### Phase I or baseline strategy (from October 1, 2015, to March 27, 2019)

In the first phase, new patients’ testing included ABO/Rh blood typing and an initial Ab screen performed by gel agglutination in a microcolumn via ProVue (Ortho-Clinical Diagnostics) from 2016 to March 2018 and Erytra (Grifols Diagnostic Solutions) afterward. The patient’s drug history was confirmed via electronic medical records. Positive Ab screen by gel method was further verified by a polyethylene glycol (PEG) IAT using 3% screen cells from Immucor with concomitant autocontrol testing. If the Ab screen was confirmed positive, the patient plasma was tested against the same lot of the Ab screen reagent cells treated with 0.2 mol/L DTT. In addition, patients who had not been transfused with RBCs in the prior 3 months underwent extended phenotype testing using monoclonal reagent antibodies for C, c, E, e, K, Jka, Jkb, S, s, and polyclonal reagent antisera for Fya, Fyb, and k (for K-positive patient) antigens. Testing reagent antibodies were procured from Bio-Rad Medical Diagnostics GmbH, Ortho-Clinical Diagnostics, and Immucor. Human erythrocyte antigen (HEA) genotype-predicted RBC phenotyping for Rh, Kell, Kidd, Duffy, and MNSs was performed at the American Red Cross Molecular Lab for patients transfused within the past 3 months. For subsequent testing on DARA patients, manual tube testing was performed by the PEG-IgG method with autocontrol, followed by repeat Ab screen with 0.2 mol/L DTT-treated cells from the same lot to detect possible underlying alloantibodies to common RBC antigens. The repeat testing for subsequent patients was performed every 7 days. Thus, the minimum time after transfusion that a new Ab screen was performed was 7 days.

### Phase II: Optimized strategy: from March 2019 to January 2025

In phase II, we optimized the testing by improving communication with the clinical team, performing direct manual tube testing by the PEG-IgG method with autocontrol, and repeating the Ab screen against 0.2 mol/L DTT-treated cells validated for a 7-day shelf-life. The standard operating procedure (SOP) for transfusion management of patients treated with DARA, including the clinical team notification process, is described in **Appendix S1**. The DTT-treated reagent red cell validation plan and results are described in **Appendix S2**. The quality controls of all testing throughout the study period were appropriate. In this phase, only Kell typing was performed for new DARA patients, and if patient's RBCs were positive for the K antigen, typing for k was also performed. Subsequent patients were treated similarly, except that the RBC phenotype was not repeated.

### Blood selection for transfusion

In phase I, ABO/Rh-compatible and Rh(D) and Kell antigen-matched RBCs were selected for transfusion. In contrast, in phase II, ABO/Rh(D)-compatible and K antigen-matched RBCs were selected. Serologic crossmatch was performed in both phases via tube testing by the PEG-IgG method for patients with no historical or current alloantibody detected, with a turnaround time (TAT) of 30 minutes and reactivities expected to match the initial PEG-IgG Ab screen reactivity strength. In phase II, the K-negative RBC inventory was improved in preparation for supporting these patients, reducing the time to type donor units. Patients were transfused with least-incompatible RBC units in both phases.

### Cost and statistical analysis

The cost value of reagents and tests was extracted from existing blood bank administrative data.

Descriptive statistical methods to report measures, such as counts, percentages, medians, and ranges, were applied using RStudio (Version 1.1.463). Comparison of control and DARA group data was performed via Fisher exact or Pearson χ^2^ tests with a statistical significance level set at *P* < .05.

## RESULTS

### Patients

Of the 850 patients with MM who were treated with DARA during the study period, 172 (20%) received RBC transfusions from October 1, 2015, to January 30, 2025. Patients’ demographics, blood testing results, and alloimmunization rate of the DARA-transfused group were compared with those of a 172-patient control group who received RBC transfusions within the same period. No patients were excluded from the study. The demographics of both groups are summarized in **Table 2**. Of 159 patients with known transplant status in the DARA group, 97 (60%) were transplanted with autologous stem cells, and a minority (3%) received other forms of transplant (allogeneic, T-cell depleted cord blood, or multiple transplants).

**Table 2:** Demographic Data

Characteristic	DARA n = 172	Control n = 172	*P* Value
Encounters, No.			
Mean ± SD/patient	6.5 ± 7.7	6.6 ± 12	.93
Range	1-56	1-68	
Sex			
M:F ratio	1.09 (90:82)	1.26 (96:76)	.58
Ethnicity, No.			
Hispanic	22	19	.91
Non-Hispanic	147	139	
Decline/unknown	3	14	
Age, y			
Median	71	72	.30
Range	26-91	33-98	

Abbreviation: DARA, daratumumab.

### Blood testing results

Blood typing results for the DARA and control groups are summarized in **Table 3**. There was no statistically significant difference between blood type distribution (χ^2^ = 8.03, *df* = 7, *P* = .32).

**Table 3:** Blood Typing Distribution Rates in the DARA and Control Groups

Blood type	DARA, %	Control, %
O POS	40	44
A POS	32	34
B POS	12	11
O NEG	6	3
AB POS	5	2
A NEG	3	5
AB NEG	1	0
B NEG	1	1

Abbreviations: DARA, daratumumab; NEG, negative; POS, positive.

Ab screen in the control group showed a 6.3% alloimmunization rate (11/172 patients tested) with the following antibodies being identified: antibody of undetermined specificity (AUS; 3 cases), anti-D (1 case), anti-E and AUS (1 case), anti-Kell (1 case), anti-Lea (1 case), anti-Leb (1 case), warm autoantibody (1 case), cold autoantibody (1 case), and high-titer, low-avidity antibody (1 case). Ab screen in the DARA group before drug administration revealed a 5.2% alloimmunization rate (9/172 patients tested) with the following antibodies being identified: AUS (5 cases), anti-C and AUS (1 case), anti-Kell (1 case), anti-Jkb (1 case), and anti-Fya (1 case). All patients (100%) developed positive Ab screens after the initial DARA dose with the expected panagglutinin pattern. By the 0.2-mol/L DTT method, the previously identified alloantibodies were demonstrated in the post-DARA testing (1 case with anti-C and AUS, 1 case with anti-Kell, 1 case with anti-Jkb, 1 case with anti-Fya) with an overall alloimmunization rate of 2.3% (4/172) and no new alloantibodies identified. Excluding AUS, the alloimmunization rate in the test group was identical pre- and post-DARA administration (2.3%).

The alloimmunization rates in the control group, pre- or post-DARA administration, were not statistically different (*P* > .05) Table 4. The RBC serology complete phenotype and genotype were performed in 46 and 19 patients. Rh/Kell serologic typing was done in 1 case, K typing in 98 instances, and Kk typing in 3 cases; genotyping found K+ k+ only in 4 cases.

**Table 4:** Alloimmunization Rates in the Control Group and the DARA Group Before and After Drug Administration

Testing phase	Alloimmunization rate, %	No. of patients (n/172)	Identified antibodies
Control group	6.3	11/172	AUS (3), anti-D (1), anti-E and AUS (1), anti-Kell (1), anti-Lea (1), anti-Leb (1), WAA (1), CAA (1), HTLA (1)
Pre-DARA (baseline)	5.2	9/172	AUS (5), anti-C and AUS (1), anti-Kell (1), anti-Jkb (1), anti-Fya (1)
Post-DARA (after a 0.2-mol/L DTT treatment)	0	0/172	No newly formed alloantibodies (only preexisting ones detected)

Abbreviations: AUS, antibody of uncertain significance; CAA, cold autoantibody; DARA, daratumumab; DTT, dithiothreitol; HTLA, high-titer, low-avidity antibody; WAA, warm autoantibody.

### Testing volumes and impact of testing strategy optimization on total testing time, turnaround time, and cost

In total, 572 and 1754 Ab IDs were performed in phases I and II, respectively, reflecting the increased patient volume. In phase I, 572 DTT treatments at 0.2 mol/L were performed with each Ab ID for a ratio of 1:1. In phase II, a total of 252 DTT red cell preparations at 0.2 mol/L were conducted, with 1 per week used for an average of ~7 patients tested with the same lot. The total time needed for performing the 572 DTT treatments at 0.2 mol/L in phase I was 858 hours, whereas the total time required for 252 DTT red cell preparations at 0.2 mol/L for the 1754 Ab IDs in phase II was 378 hours. The estimated time reduction for the 0.2-mol/L DTT red cell preparation was from 90 minutes to 9 minutes per patient. The turnaround time (TAT) for pretransfusion testing (excluding crossmatch) using the 0.2-mol/L DTT preparation of reagent red cells for each patient was 2.5 hours for new patient testing and remained the same for each subsequent testing in phase I. Using the 7-day validated 0.2-mol/L DTT-treated red cells led to a TAT reduction to 1.25 hours for new patients and 1 hour for subsequent testing in phase II.

In addition, in phase I, the cost of testing for phenotype and genotype (45 phenotype and 19 genotype cases) was determined to be $13 253 (estimated at $206 per patient), whereas in phase II, for the 98 patients serologically tested for K (and, if positive, for k antigens), the total reagent cost was $1 per patient. The impact of strategy optimization is summarized in **Table 5**.

**Table 5:** Summary of Intervention Strategies and Their Impact

Intervention	Impact
Prenotification	Allowed skipping the automated gel testing, which decreased the TAT by 1 hour
7-day validated 0.2-mol/L DTT reagent red cells	On average, ~7 Ab screens were tested with the same validated 0.2-mol/L DTT reagent red cells per week Testing time reduced from 90 to ~9 min/patient
Determination of K(k) phenotype rather than full phenotype or genotype	Reduced the pretransfusion cost from $146 (full phenotype) or $353 (genotype) to $1-10/patient K(k) testing via serology

Abbreviations: DARA, daratumumab; DTT, dithiothreitol; TAT, turnaround time.

## DISCUSSION

Pretransfusion testing in patients with MM and DARA administration is known to be laborious and costly. Notification by the clinical team is a critical step, and 1 of the first interventions was establishing robust communication with the clinical team. This was supported by including a history check in the SOP and electronic notification processes from the pharmacy.^9^

Detection of potentially underlying alloantibodies is mitigated in most institutions using the Ab screen with 0.2-mol/L DTT-treated reagent red cells, disrupting the CD38-DARA bond on the RBC surface. In our study, the minimum time after transfusion at which a new Ab screen was performed was 7 days, and all patients underwent a new Ab screen. Also, the Association for the Advancement of Blood and Biotherapies (AABB), formerly known as American Association of Blood Banks, recommends that these patients have their RBC phenotype determined before DARA initiation or genotype is performed, which has become the standard in pretransfusion testing.^10^ Although this is a 1-time event, it adds to the total cost ($13 399). However, knowing a patient’s phenotype is a safety net in cases when transfusion is necessary and complete, Ab ID cannot be performed. In these cases, providing phenotypically matched RBCs is the safest course of action to prevent potential hemolytic transfusion reactions.

Some institutions perform a 0.2-mol/L DTT reagent preparation with each testing and/or transfusion of phenotypically matched RBCs. We found that unnecessary.

Numerous studies, including ours, have shown that the alloimmunization rate in this category of patients is zero or comparable with general alloimmunization rates, as outlined in **Table 6**, which summarizes significant studies investigating this issue.

**Table 6:** Comparison of Study Characteristics and Alloimmunization Rates in Patients Administered Anti-CD38 vs Those Found in the Current Study

Study	Patient No.	Study duration	History of RBC transfusion	Control group	Alloimmunization rate, %
Anani et al, 2017^11^	62	7 mo	Yes	No	0
Bub et al, 2017^12^	5	1 y	Yes	No	0
Chari et al, 2017^13^	124	NA	Yes (in 38%)	No	0
Carreño-Tarragona et al, 2018^14^	33	NA	Yes	No	0
Deneys et al, 2018^15^	14	NA	Yes (in 79%)	No	0
Cushing et al, 2019^5^	91	1 y	Yes	No	3
Ye et al, 2019^6^	45	3.5 y	Yes	Yes	0
Solves et al, 2020^16^	44	2.5 y	Yes	No	0
Phou et al, 2020^17^	52	1.1 y	Yes	No	0
Tauscher et al, 2021^7^	145	3.25 y	Yes	No	4.1
Bullock et al, 2021^8^	341	0.33 y	No	No	0.4
Safić Stanić et al, 2024^18^	68	5 y	Yes (in 56%)	No	0
Current study	172	>9 y	Yes	Yes	0

Abbreviations: NA, not applicable; RBC, red blood cell.

Posttransfusion Ab screen in the DARA group revealed no additional clinically significant alloantibodies. Bullock et al^8^ reported that in 80% (21/26) of patients with an underlying antibody, the antibody was identified in samples before DARA initiation. However, in his study, the alloimmunization rates post-DARA are reported without confirmation of transfusion history and, therefore, are likely overestimated. Interestingly, the overall alloimmunization rate was lower in the post-DARA group, with identical antibody profiles for clinically significant alloantibodies. This difference is due to the absence of AUS reactivity in the post-DARA group. The reason for this finding is unknown as it is the cause for the AUS reactivity. AUS reactivity has been previously observed to disappear or develop into an identifiable pattern of a clinically significant alloantibody.^19^ In this study, all AUS reactivity disappeared. It is possible that the AUS reactivity was no longer detectable with the Ab screen using 0.2-mol/L DTT reagent cells due to antigen modification and/or changes induced in the antigen-antibody reaction. Reduced efficacy of 7-day DTT could have also been a contributing factor. However, this is less likely given that all controls were adequate.

Truly, the etiology of AUS reactivity is not defined, and this term represents a consensually accepted nomenclature for what is a nonspecific reactivity. More studies should aim to define and understand AUS reactivity’s etiology further.

This study highlights a potential new strategy for pretransfusion testing in patients administered DARA and undergoing transfusion. This strategy implies validating the 0.2-mol/L DTT reagent red cells used for Ab screen testing for 7 days, which decreases the testing time to 17% (from 90 to 15 minutes per patient). As a result, the total TAT for pretransfusion testing (excluding crossmatch) can be reduced to 1.25 hours for a new patient and 1 hour for a known patient, representing a reduction to less than one-third of the routine TAT for these patients. This is especially important when increasing testing volumes. This study shows that 3 times more Ab IDs were performed in less than half the time initially required for pretransfusion testing using this algorithm. The pretransfusion testing cost is 100 times less than those previously reported. In addition, this algorithm shows that testing for K antigens and providing K-matched RBCs rather than phenotypically matched RBCs is safe, as these patients do not develop new alloantibodies. This is not surprising considering the alloimmunization rates described in the literature.^6,18,16^ A similar strategy is recommended for alloimmunization prophylaxis in frequently transfused patients with sickle cell disease.^20^ It is feasible, then, to propose that providing ABO/D-compatible RBCs matched for Kell may reduce the transfusion-associated cost without jeopardizing patient safety in this category of patients. Providing extended antigen-matched RBC units beyond ABO, D, and Kell as routine might not be necessary when patients had negative Ab screen results using 0.2 mol/L DTT-treated cells.

Although this study provides a practical solution for blood banks, certain limitations should be acknowledged. Factors such as patient variability and reagent sensitivity may impact the strategy’s effectiveness in different settings. Future research should assess its cost-effectiveness in diverse clinical environments and identify potential barriers to widespread adoption. While the results demonstrate a reduction in testing time and cost, further studies should explore the long-term effects of DARA on alloimmunization and transfusion safety. Additionally, the applicability of these findings to other patient populations, such as those receiving different immunosuppressive therapies, should be investigated.

Comparing this optimized approach with other methods for mitigating DARA interference would help confirm its accuracy, cost, and efficiency benefits.

## CONCLUSIONS

In conclusion, this is the most extensive study on patients treated with DARA who were transfused, demonstrating no increased alloimmunization risk. In addition, the study proposes a strategy for mitigating DARA interference that leads to a more cost-effective pretransfusion testing approach.

## Supplementary material

Supplementary material is available at *American Journal of Clinical Pathology* online.

aqaf060_suppl_Supplementary_Appendix_S1

aqaf060_suppl_Supplementary_Appendix_S2
