# Supplementary material for: Optimized strategy to mitigate daratumumab interference in blood bank testing: Reducing cost and time
Source: Am J Clin Pathol. 2025 Jul 2;164(3):283–8. doi: 10.1093/ajcp/aqaf060 (PMC12421236; doi:10.1093/ajcp/aqaf060)
Supplement: aqaf060_suppl_Supplementary_Appendix_S1 [file aqaf060_suppl_supplementary_appendix_s1.pdf]

Appendix 1

STANDARD OPERATING PROCEDURE FOR TRANSFUSION MANAGEMENT OF PATIENTS TREATED WITH DARATUMUMAB, ISATUXIMAB, AND OTHER ANTI-CD38 DRUGS

I. PURPOSE

To outline the transfusion management approach (identification of patients receiving the drug, pretransfusion testing and selection of blood products) for patients treated with daratumumab (DARA), isatuximab (SAR), or other anti-CD38 drugs. These drugs are commonly used to treat patients with multiple myeloma (MM). Because CD38 is also expressed in RBCs, DARA binds RBCs and interferes with antibody screening, antibody identification, and compatibility testing. DARA-mediated positive indirect antiglobulin test may persist for up to 6 months after cessation of therapy. Other anti-CD38 drugs like SAR are also known to cause similar interference.

II. EQUIPMENT & MATERIALS

1. Reagent red cells
2. Anti-IgG (IgG)
3. Polyethylene glycol (PEG)
4. 0.2M Dithiothreitol (DTT)-treated reagent cells
5. Coombs control check cells
6. Blood Bank buffered saline
7. Cell washer

8. Calibrated centrifuge
9. Agglutination viewer
10. Microscope
11. Laboratory Information System (LIS)
12. Hospital Information System (HIS)
13. 10 X 75mm test tubes
14. Transfer pipette
15. 37°C heat block incubator
16. Antigram and Antibody Identification Summary Sheet

III. QUALITY CONTROL

1. Reagents must pass daily quality control (QC) testing on the day of use.
2. Coombs control cells must react as expected for negative reactions to be valid.
3. 0.2 M DTT-treated reagent cells must pass QC testing before use.
4. The Immunohematology Specialist or designee reviews workups for completeness and accuracy.

IV. PROCEDURE

A. Identification of DARA patients or patients receiving SAR or other anti-CD38 drugs.

1. Patients on DARA, SAR, or other anti-CD38 drugs may initially come to Transfusion Service's attention because serologic testing demonstrates weak reactivity at the antiglobulin phase with most reagent cells or shows a panagglutinin pattern, with/without a positive auto control/DAT.

2. Check the patient’s diagnosis in HIS. If the patient has MM, check for DARA or SAR history.

3. If unable to verify drug history in HIS, call the provider and ask if the patient is currently on DARA, SAR, or other anti-CD38 drugs or has received one of these drugs in the past six months.

4. The clinical team notifies (via phone call) the Transfusion Service of new patients receiving DARA, SAR, or other anti-CD38 drugs. Record the patient’s name, MRN, name of drug, start date, and caller’s name and date. Ask the MD to order an initial Type & Screen before treatment with one of these drugs. Notify the Immunohematology Specialist.

5. Oncology service and Pharmacy may directly notify (via email) the Transfusion Service Supervisor/Immunohematology Specialist/MD about new patients receiving or scheduled to receive DARA, SAR, or other anti-CD38 drugs.

B. Update the patient’s Blood Bank Administrative Data (BAD) with the following information in the “Comments” field:

1. Anti-CD38 patient, starting or receiving DARA/SAR/other anti-CD38 (drug) on MM/DD/YY.
2. Add code “RIRR” to transfusion attributes.
3. The hospital name if the patient has received this drug outside of UCSF.
4. The MD/RN name and date of notification.

C. Perform baseline testing before testing with DTT-treated reagent cells:

1. Perform a Type and Screen.
2. Obtain a full transfusion history, regardless of antibody screen results and a history of antibodies and phenotype from outside hospitals.

3. Type for K antigen if the patient has not received RBC transfusions in the past 8 weeks. If K antigen is typed positive, type for k antigen. Update the BAD file for "Requires K negative" based on the patient's K antigen status. If there is difficulty interpreting antigen typing results, please ask the Immunohematology Specialist to review them.

4. A genotype may be performed on recently transfused patients if indicated.

D. Antibody identification:

1. If the initial positive antibody screen was done using the gel method, perform the antibody screen using the PEG-IgG method.

2. DARA typically shows a weak panagglutinin pattern on the PEG antibody screen. SAR and other anti-CD38 drugs may show a weak panagglutinin pattern or may not react with all cells.

3. If two screening cells are non-reactive by PEG-IgG method: Run a standard antibody identification panel and auto control by PEG-IgG method to rule out underlying alloantibodies.

4. If the initial PEG antibody screen shows the typical weak panagglutinin pattern that is seen with DARA interference, test 0.2 M DTT treated same lot screen cells by PEG-IgG method to rule out common clinically significant red cell alloantibodies. Verify that the batch-treated reagent cells show no or only slight hemolysis before the testing and document on the QC Log for 0.2 M DTT-Treated Reagent cells.

5. Asymmetric or variable strength reactivity or stronger than usual reactivity suggests the presence of underlying alloantibodies. Test additional DTT-treated selected cells to rule out or identify antibodies.

6. If the autocontrol is positive, perform a direct antiglobulin test (DAT) and elution. Test the eluate against both 'DTT-treated' and 'untreated' screening cells (test against group A and B cells if indicated).

7. If you suspect that the patient on DARA has warm autoantibody (eg, after DTT treatment, panel reactivity does not go away, and eluate is reactive with both non-treated and DTT-treated screening cells), or you suspect that the patient has alloantibody against a high-frequency antigen (panel reactivity does not go away after DTT treatment), please consult Immunohematology Specialist and/or Transfusion Service MD. Such cases are handled on a case-by-case basis.

8. Send the sample to the Blood Supplier Reference Lab if the antibody identification can't be completed promptly due to limited resources for patients needing RBC transfusion urgently.

E. Selection of RBCs for transfusion:

1. Provide K negative units if the patient typed K negative.

2. If the patient is negative for high-frequency antigens in the Kell blood group, like k, Kpb, or Jsb, consult an Immunohematology Specialist and/or Transfusion Service MD for blood selection.

3. If a transfusion is required urgently before the completion of antibody identification, emergency release procedures should be followed.

F. Compatibility Testing:

1. Perform crossmatches using the PEG-IgG method.

2. Compatibility testing is expected to show similar reaction strength to panel cells. At least incompatible units may need to be issued. All units should be compatible if a current sample shows no interference from an anti-CD38 drug or warm autoantibody.

G. Post-DARA Patient Support Updates

1. Identifying Post-DARA Patients:

a. Suspect a patient discontinued DARA treatment when three consecutive samples were taken with a negative antibody screen.

b. Discontinuation of DARA is confirmed with the provider or nurse (do not solely rely on Apex notes)

2. BAD File Update Process:

a. Update BAD fields as follows:

- Antibody Screen Summary: Change to NEGATIVE
- Antigen/Antibody: Remove PAN, Remove RKEL (if applicable)
- Comments: Add a note explaining the removal of DARA's requirements. Example: 3X negative screens, nurse Jane Doe confirmed the discontinuation of DARA. Tech initials/Date
- Retain all other DARA-related comments, such as K typing results.

b. Responsibility and Delegation:

- Any Clinical Laboratory Scientist (CLS) may perform this update.
- Depending on workload, requests for updates can be forwarded to the Immunohematology Sr Specialist, Bench Specialist, or Supervisor.

Note: If a patient alternates on and off DARA treatment, re-enter information into the BAD file

V. NOTES

A. Agglutination due to anti-CD38 drugs may occur in all media (low ionic strength saline, polyethylene glycol) and manual and automated methods.

B. DTT-treated cells can eliminate the interference for antibody screening and identification.

C. Because DTT treatment destroys Kell antigens, K-negative units should be provided to K-negative patients. K-negative units should be provided to K-negative patients unless anti-k has been ruled out.

D. Antibodies against other DTT-sensitive blood group antigens like anti-Yta, anti-JMH, antibodies in the Knops group, antibodies in the Dombrock group, and some examples of anti-Vel will not be detectable when tested against 0.2 M DTT-treated cells.
