# Supplementary material for: Optimized strategy to mitigate daratumumab interference in blood bank testing: Reducing cost and time
Source: Am J Clin Pathol. 2025 Jul 2;164(3):283–8. doi: 10.1093/ajcp/aqaf060 (PMC12421236; doi:10.1093/ajcp/aqaf060)
Supplement: aqaf060_suppl_Supplementary_Appendix_S2 [file aqaf060_suppl_supplementary_appendix_s2.pdf]

**Appendix S2: Process Validation Plan**

## A. VALIDATION OF 0.2M DTT (DITHIOTHREITOL) TREATED REAGENT CELLS FOR TESTING UP TO 7 DAYS.

### I. PURPOSE

To verify the blood group, antigens on reagent red cells are affected as expected after the reagent red cells are treated with 0.2 M DTT (dithiothreitol) for up to 7 days. 0.2 M DTT-treated reagent cells within 7 days serve to detect or rule out common red cell antibodies in the Rh group, Duffy group, Kidd group, and MNS group.

### II. DESCRIPTION

0.2M DTT treatment destroys antigens in the Kell system and some other minor blood groups, while the antigens in the Rh, Duffy, Kidd, and MNS groups are unaffected. Interference by anti-CD38 drugs, like Daratumumab (DARA) in the indirect antiglobulin test can be abolished by testing 0.2M DTT-treated reagent cells because DTT disrupts DARA binding by cleaving CD38 from the red cells.

### III. MATERIALS

3% Reagent red cells

0.2M DTT

Antisera

Alsever solution

Calibrated centrifuge / Cell washer

Agglutination viewer

Microscope

10 X 75mm test tubes

Disposable plastic transfer pipette

Anti-Human IgG Globulin (IgG)

Polyethylene Glycol (PEG)

Coombs control cells (IgG)

Blood Bank buffered saline

Haemonetics color comparator

IV. PROCEDURE

1. Select one set of 3% reagent screen cells that are 2 or 3 weeks in the inventory but will not expire within the next 10 days.
2. According to the current SOP, the reagent screen cells are treated with 0.2M DTT.
3. Centrifuge the 0.2M DTT-treated reagent cells, remove saline and re-suspend the DTT-treated cells in Alsever solution. Label the tube/bottle as 0.2M DTT treated cells in Alsever solution and keep it in the reagent refrigerator.
4. Observe for hemolysis. Perform hemolysis check before treatment, on day 1, day 5, and then on day 7 after the treatment.
5. Perform antigen typing for C, E, c, e, K, k, $Fy^a$, $Fy^b$, $Jk^a$, $Jk^b$, M, N, S, s, $Le^a$, $Le^b$, and $P_1$ antigens before treatment on day 1 and then day 7 after the treatment.

6. Test known samples with red cell antibodies by the PEG-IgG method as described in **Table S1**:

**Table S1** Antigen testing summary for 0.2M DTT-treated reagent red cells.

| Samples from patients | Prior to treatment (untreated cells) | Day 1 after treatment | Day 5 after treatment | Day 7 after treatment |
|---|---|---|---|---|
| DARA patient | √ | √ | √ | √ |
| anti-C | √ | √ | √ | √ |
| anti-c | √ | √ | √ | √ |
| anti-$Fy^a$ | √ | √ | √ | √ |
| anti-S | √ | √ | √ | √ |
| anti-K | √ | √ | √ | √ |

Abbreviations: DARA, daratumumab; DTT, dithiothreitol.

7. Observe for cloudiness

8. Perform a cloudiness check before treatment, on day 1, day 5, and then on day 7 after the treatment.

   Note: On day 1 and after, wash the cells two to three times with saline to remove Alsever's solution, then make a 3% cell suspension for testing.

9. Submit results and worksheet to the Immunohematology Specialist for review.

V. EXPECTED OUTCOME

1. Over 7 days, all present antigens, except K antigen, are expected to remain on the DTT-treated reagent cells without or with slight hemolysis present. Over 7 days, cloudiness will not be present.

1. Interference by anti-CD38 drugs will be abolished after the DTT treatment up to day 7.

2. Anti-C, -c, -$Fy^{a,}$ and anti-S from patients are still present and detectable on the DTT-treated cells until day 7.

3. Anti-K from the patients was only detected on the untreated cells and not detectable on the DTT-treated cells until day 7.

## V. OUTCOME FAILURES

1. If antigens on reagent cells do not remain intact on day ten or the batch is noted to show excessive marked hemolysis before day 7.
2. If the antibodies failed to react as expected (listed above).

## VI. CRITERIA FOR ACCEPTABILITY

1. The antigens in the Rh blood group, Duffy group, Kidd group, MNS, Lewis, and $P_1$ group are unaffected by the DTT treatment up to day 7. Variation of testing strength is 1+ or less during this period compared to testing results obtained before the DTT treatment.
2. K antigen becomes negative after 0.2M DTT treatment up to day 7.
3. No marked hemolysis is observed on day 7.
4. Interference by anti-CD38 drugs will be abolished after the DTT treatment up to day 7.
5. Anti-C, -c, -$Fy^{a,}$ and anti-S from patients are still present and detectable on the DTT-treated cells until day 7.
6. Anti-K from the patients was only detected on the untreated cells and not detectable on the DTT-treated cells until day 7.
7. Over 7 days, cloudiness will not be present.

**REVIEW AND APPROVAL FOR PROCESS VALIDATION PLAN:**

**The Process Validation plan is: _______approved**

**_______rejected**

________________________________ _________________

Author Date

________________________________ _________________

Sr. Supervisor Date

________________________________ _________________

Medical Director Date

______________________________ _________________

Compliance Officer Date

**The validation results are: _______approved**

**_______approved with limitations**

**_______rejected**

**Limitations:**

________________________________ _________________

Author Date

________________________________ _________________

Sr. Supervisor Date

________________________________ _________________ Medical Director

Date

________________________________ _________________

Compliance Officer Date

**SUMMARY OF VALIDATION RESULTS**

**FOR 0.2 M DTT-TREATED REAGENT RED CELLS FOR TESTING UP TO 7 DAYS**

I. TIMEFRAME

The validation process for using 0.2M DTT-treated reagent red cells was performed between 10/9/18 and 10/16/18.

II. RESULTS

The following results were obtained:

1. The antigens carried on the reagent cells in the Rh blood group, Duffy group, Kidd group, MNS, Lewis, and $P_1$ group are not affected by the DTT treatment up to day 7.
2. Variation of antigen strength is less than 1+ during this period. K antigen becomes negative after 0.2M DTT treatment up to day 7.
3. No marked hemolysis is observed on day 7 (using Haemonetics color comparator reading of no greater than 2).
4. No cloudiness was observed in 0.2 M DTT-treated cells for up to 7 days.
5. Interference by DARA was abolished after the DTT treatment up to day 7.
6. Anti-C, -c, -$Fy^{a,}$ and anti-S from patients were detected as expected on the DTT-treated cells up to day 7.
7. Anti-K from the patients was detected only on the untreated cells and not on the DTT-treated cells up to day 7.

III. CONCLUSION

0.2 M DTT-treated reagent red cells can be used for testing for up to 7 days to detect or rule out possible red cell antibodies against common red cell antigens except for antibodies within the Kell blood group.
